# Toxigenic properties and *stx* phage characterization of *Escherichia coli* O157 isolated from animal sources in a developing country setting

**DOI:** 10.1186/s12866-018-1235-3

**Published:** 2018-08-31

**Authors:** Mahdia Rahman, Ashikun Nabi, Md Asadulghani, Shah M. Faruque, Mohammad Aminul Islam

**Affiliations:** 1 Enteric and Food Microbiology Laboratory, Laboratory Sciences and Services Division (LSSD), International Centre for Diarrhoeal Disease Research, Bangladesh (icddr,b), Mohakhali, Dhaka, 1212 Bangladesh; 2 Present Address: Department of Biology, University of Vermont, Burlington, VT 05405 USA; 3 Present Address: Department of Mathematics and Natural Sciences, BRAC University, Mohakhali, Dhaka, 1212 Bangladesh

**Keywords:** *E. coli* O157, Shiga toxin 2, Toxin non-producing, *stx* phage

## Abstract

**Background:**

In many Asian countries including Bangladesh *E. coli* O157 are prevalent in animal reservoirs and in the food chain, but the incidence of human infection due to *E. coli* O157 is rare. One of the reasons could be inability of the organism from animal origin to produce sufficient amount of Shiga toxin (Stx), which is the main virulence factor associated with the severe sequelae of infection. This study aimed to fill out this knowledge gap by investigating the toxigenic properties and characteristics of *stx* phage of *E. coli* O157 isolated from animal sources in Bangladesh.

**Results:**

We analysed 47 *stx*_2_ positive *E. coli* O157 of food/animal origin for *stx*_2_ gene variants, Shiga toxin production, presence of other virulence genes, *stx* phage insertion sites, presence of genes associated with functionality of *stx* phages (*Q*_933_ and *Q*_21_) and *stx*_2_ upstream region. Of the 47 isolates, 46 were positive for both *stx*_2a_ and *stx*_2d_ while the remaining isolate was positive for *stx*_2d_ only. Reverse Passive Latex Agglutination assay (RPLA) showed that 42/47 isolates produced little or no toxin, while 5 isolates produced a high titre of toxin (64 to 128). 39/47 isolates were positive for the Toxin Non-Producing (TNP) specific regions in the *stx*_2_ promoter. Additionally, all isolates were negative for antiterminator *Q*_933_while a majority of isolates were positive for *Q*_21_ gene suggesting the presence of defective *stx* phage. Of the *yehV* and *wrbA* phage insertion sites, *yehV* was found occupied in 11 isolates while *wrbA* site was intact in all the isolates. None of the isolates was positive for the virulence gene, *cdt* but all were positive for *hlyA*, *katP*, *etpD* and *eae* genes. Isolates that produced high titre Stx (*n* = 5) produced complete phage particles capable of infecting multiple bacterial hosts. One of these phages was shown to produce stable lysogens in host strains rendering the Stx2 producing ability.

**Conclusion:**

Despite low frequency in the tested isolates, *E. coli* O157 isolates in Bangladesh carry inducible *stx* phages and have the capacity to produce Stx2, indicating a potential risk of *E. coli* O157 infection in humans.

## Background

Gastrointestinal infection caused by virulent *Escherichia coli* O157 in humans can lead to haemorrhagic colitis and often to a more serious complication, known as haemolytic–uremic syndrome. Cattle are asymptomatic reservoirs for *E. coli* O157 [1–3]. Food products contaminated by cattle feces (either direct or indirect contamination) are the cause of numerous outbreaks [2, 4, 5].

Shiga toxins (Stx1 and Stx2) are the important virulence factors of *E. coli* O157 that play a crucial role in the progression of the most severe form of the disease in human. Epidemiological data suggest that Stx2-producing *E. coli* O157 isolates are more commonly associated with serious diseases than isolates producing Stx1 [6]. *stx* phages are temperate lambdoid bacteriophages and Shiga toxin genes are located in the late–phase region, downstream of *Q* homologue and upstream of genes *S*, *R* and *Rz*, required for the release of phage particles [7]. Furthermore, *stx* phages are heterogeneous in nature since they differ in their DNA structures, PFGE pattern, virion morphology and spectra of hosts [8]. DNA damaging agent such as UV-light and certain antibiotics that effect DNA synthesis, such as mitomycin C and trimethoprim-sulfamethoxazole, can provoke the induction of phages [9]. Induction of lysogenized phages eventually leads to the lysis of host bacterial cell and deliverance of free infectious phage particles [10]. These free phage particles play an important role in the transduction of *stx* genes to susceptible bacteria and thus can lead to the emergence of new Shiga toxin-producing *E. coli* (STEC) pathogens by horizontal gene transfer [11].

Although *E. coli* O157 is frequently isolated from cattle, with a prevalence varying from 0 to 41.5% [1, 12], the human infections are relatively uncommon despite the low infectious dose of the organisms [13, 14]. In Bangladesh, there is a high prevalence of *E. coli* O157 in slaughtered animals especially in buffalo and cow [15]. Raw meats from these animals were also found contaminated with *E. coli* O157 [16]. Although diarrheal stool samples are not routinely tested for *E. coli* O157 in Bangladesh, several studies have indicated a very low prevalence of this organism among patients with diarrhea [17]. Several studies reported the similar trend of data in other Asian countries including Thailand and Malaysia [18, 19].

A previous study has shown that *E. coli* O157 isolates from bovine and human origin are distributed among two separate genetic lineages and the isolates of bovine lineage are less virulent or not capable of transmitting to humans [20]. In general, bovine originated O157 isolates produce a low amount of Shiga toxins as compared to virulent strains isolated from human [21, 22]. In Asian countries, one common reason for the low or no toxin production capacity of bovine *E. coli* O157 isolates is linked to the presence of genetically defective *stx* phages. Previous study has demonstrated that the defective phages carry mutations in the promoter region of the *stx*_2_ gene [23]. The defective promoter region and the absence of bacteriophage antiterminator gene *Q*_933_ in the integrated phage genome inhibit the expression of *stx*_2_ genes in these strains. The product of *Q*_933_ gene acts at *qut* (Q utilization sequence) and promotes the transcription of terminators, *t*_*R’*_ that activates the phage late promoter *p*_*R’*_, which is essential for strong *stx*_2_ transcription and is associated with higher Shiga toxin production [23]. In *stx*_2_ phage genome, the *stx*_2_ gene is located downstream of *Q* gene. To identify the defective upstream regions of *stx*_2_ gene expanding from *q* to *stx*_2_, PCR primers were designed from *Q* gene and its flanking region and these PCR assays have been used as diagnostic tools for identification of toxin-non-producing (TNP) strains of *E. coli* O157 [12, 23]. A similar PCR assay named as NM-PCR has been developed by Taylor et al. for detection of the region from *Q* to *stx*_2_ gene [24].

We hypothesize that *E. coli* O157 strains from animal origin in Bangladesh are less virulent to cause infection in humans, however the potential for enhanced pathogenicity and evolution of STEC strains cannot be ruled out, given that predicted climate changes associated with adverse natural events in this region. In this study, we analysed a collection of *E. coli* O157 strains isolated from slaughtered animals and raw meats in Bangladesh for their ability to produce Shiga toxin. *E. coli* O157 isolates with varying range of toxigenicity were further analysed for induction and propagation of the lysogenic *stx* phage, as well as the genetic diversity of the phages.

## Methods

### Bacterial strains

A total of 47 *E. coli* O157 isolates were included in this study. Of these 40 were isolated from fecal samples from 174 buffalo, 139 cows, and 110 goats at a slaughterhouse in Dhaka of the Mohakhali area in Bangladesh during the period from January to May 2006. The remaining 7 samples were isolated from 90 raw meat samples from the same slaughterhouse during the period from July 2006 to January 2007. Previous studies described details about isolation methods and sources of these strains [15, 16].

### Assays for toxin production by *E. coli* O157 isolates

Stx2 production was tested using a commercially available reverse passive latex agglutination kit (Oxoid VTEC-RPLA Toxin Detection Kit, Remel, Lenexa, KS, USA) according to the manufacturer’s instruction. Briefly, isolates were grown at 37 °C overnight on brain-heart infusion agar from which a few isolated colonies were taken to make a suspension in normal saline supplemented with polymixin B (5000 U/ml). The suspension was incubated for 30 min followed by centrifugation at 4000 rpm for 20 min. Supernatant was transferred to a 96-well V-bottom microtitre plate and two- fold serial dilutions were made with a supplied diluent. Stx2 was detected by adding test reagents to each of the wells and the plate was incubated at room temperature for 24 h. Visual appearance of agglutination indicated positive result. RPLA titre was defined by the reciprocal of the highest dilution of test sample giving a positive agglutination. *E. coli* O157:H7 NCTC 12079 and *E. coli* O157:H7 NCTC 12900 was used as positive and negative control, respectively. The sensitivity of the test is 1 to 2 ng/ml of *E. coli* Shiga toxin.

### Detection of virulence genes by PCR

Isolates were tested for the presence of virulence gene (*stx*_1_, *stx*_2_, *eae, hly*_*EHEC*_, *katP*, and *etpD* gene) by PCR following methods described earlier [16]. Isolates carrying *stx*_2_ genes were subtyped by PCR following the procedure described by Scheutz et al. [25]. Presence of cytolethal distending toxin (*cdt*) gene was detected by PCR following conditions as described [26].

### Identification of TNP strains by PCR assays

All isolates were tested by three different PCR assays such as TNP-PCR, NM-PCR and PCR for antiterminator gene alleles. DNA was extracted from bacterial cells by boiling a single colony in distilled water. In TNP-PCR assay, four separate PCR reactions (TNP-A, TNP-B, TNP-C and TNP-D) were carried out with seven primers described by Koitabashi et al. [23]. An isolate was considered TNP-PCR positive if expected amplicons were observed in all four reactions. For NM-PCR, 0.5 μM of each forward and reverse primer, NM-F and NM-R was added to mastermix [24]. Bacteriophage antiterminator gene alleles *Q*_933_, and *Q*_21_were detected by PCR as described previously [12]. *E. coli* O157:H7 NCTC 12079 was used as positive control for detection of *Q*_933_ gene.

### Determination of *stx*_2_ phage integration sites in the *E. coli* genome

The *stx*_2_ phage integration sites in *E. coli* O157 isolates were determined by PCR for *wrbA* and *yehV* gene loci and genes present in their left and right junctions following the methods described by Shaikh and Tarr [27]. Amplification of genes indicates that the insertion locus is intact and no phage is inserted while no amplification may indicate that *stx*_2_ phage is inserted and thus the site is occupied. Occupancy of a site was confirmed by detecting regions flanking the left and right junction of the phage.

### Bacteriophage induction, isolation and preparation of phage lysate

The lytic cycle of temperate bacteriophages was induced by mitomycin C (MMC) treatment as described earlier [10, 28]. Briefly, a single colony of each isolate from MacConkey agar plate was inoculated into 6 ml of LB broth supplemented with 5 mM CaCl_2_ and incubated at 37 °C for 18–24 h with shaking. The culture was adjusted to 0.1 at 600 nm optical density (OD) with the addition of fresh LB broth and incubated at 37 °C with shaking until the OD of culture reached 0.2–0.4. After incubation, culture was divided into two equal aliquots and MMC was added to one portion at a final concentration of 1.0 μg/ml. Both aliquots of culture were then incubated at 37 °C with shaking (120 rpm). Bacterial growth in two aliquots was monitored by taking OD values at 1 h interval consecutively for 10 h and phage-mediated lysis of cells was revealed by a decreasing trend of OD value at 600 nm. The entire process of phage induction and phage-mediated lysis of bacterial cells was repeated three times for each isolate [10]. OD values obtained from all three replicas of each isolates were analysed for mean and standard deviation, which were plotted against time to depict the kinetics of phage induction.

### Extraction of *stx*_2_ phage DNA

To confirm the presence of *stx*_2_ gene in phage DNA, phage DNA from the isolates was isolated from 100 ml of cultures following the procedure described earlier [28]. Briefly, bacterial isolates were grown in LB broth supplemented with MMC (1 μg/ml) for 10 h. Culture broth was centrifuged and supernatant was collected and filtered through low protein-binding 0.22 μm membrane filters. Filtered supernatant was treated with polyethylene glycol (PEG) (20%) supplemented with 10% NaCl and kept at 4 °C for overnight. Treated supernatant was centrifuged and the pellet was re-suspended in SM buffer [0.58% NaCl, 0.2% MgSO_4_.7H_2_O, 1 M Tris-Cl (pH 7.5), 0.01% gelatin]. The suspension was treated with DNase 1 (10 mg/ml) and RNase (10 mg/ml) followed by proteinase K (20 mg/ml). DNA was extracted using equal volume of Phenol: Chloroform: Isoamyl alcohol (25:24:1) followed by precipitation with double volume of absolute alcohol and 3 M sodium acetate (pH 4.6). Phage DNA was dissolved in TE buffer and stored at -20 °C until further use. *stx*_2_ gene was detected in phage DNA by PCR as described previously [16].

### Determination of genetic diversity of *stx*_2_ phages

To determine genetic diversity of the phages, DNA was subjected to Field Inversion Gel Electrophoresis (FIGE) analysis as described earlier [28]. Briefly, precipitated phage particles were dissolved in TES buffer (10 mM Tris-Cl, 10 mM MgCl_2_, 100 mM NaCl) which was embedded in plugs of 1% Certified Low Melt Agarose (Bio-Rad Laboratories, Inc., CA, USA). Plugs were treated with DNase I (1000 U/ml), RNase A (50 μg/ml) in DNase I buffer (10 mM Tris pH 7.5, 2 mM MgCl_2_, 0.5 mM CaCl_2_) for 2 h at 37 °C, followed by treatment with proteinase K (100 mg/ml) in TE buffer containing 1% SDS at 50 °C for overnight, then washed with TE buffer for three times each at 15 min interval. Then plugs were sliced into appropriate sizes, treated with *Eco*RI restriction enzyme and subjected to FIGE. FIGE was performed with 1% agarose gel in a CHEF MAPPER (Bio-Rad Laboratories) where the initial and final switch times were set at 0.11 and 0.92 s, respectively. Total run time was 19 h with 9.0 V/cm (forward) and 6.0 V/cm (reverse) at a constant temperature of 14 °C. The gel was stained with ethidium bromide to visualize DNA bands. Presence of *stx*_2_ in different phage FIGE patterns was carried out by Southern blotting using the radioactively labeled *stx*_2_ gene probe following standard methods [29]. Both TNP positive (M18, M133, M163 and G51) and negative (G71) strains producing high titre of Stx2, were analysed to find if there is any differences between phages from these two types of strains. Along with these 5 isolates, another 5 isolates (C96, M143, M168, M171, M173) with little or no toxin producing capacity were analyzed to determine if there is any association between toxin production and inducibility of phages (with and without MMC).

### Evaluation of the ability of the induced phages to infect different host strains

The following bacterial strains were used to evaluate the infectivity of inducible *stx*_2_ phages: *E. coli* K-12, *E. coli* MC1061, *E. coli* DH5α and clinical isolates of *Shigella sonnei* sh2, *Shigella flexneri* 1b 212,789, *S. flexneri* 2a 212,710, *S. flexneri* 2b KH-000151, *S. flexneri* 3a 212,670, *S. flexneri* 3b KH-000142, *S. flexneri* 4 KH-000221, *Shigella dysenteriae* 1 K-235, *S. dysenteriae* 2 K-309, *S. dysenteriae* 3 K-359, *S. dysenteriae* 4 K-1035, *Salmonella* Paratyphi b 212,693, *Vibrio cholerae* O1 206,052, enteropathogenic *E. coli* W56C3. One hundred microliter of phage lysate and 500 μl of exponential-phase culture of each host strain were mixed with 6 ml of LB soft agar (containing 0.7% agar) supplemented with 5 mM CaCl_2_ and overlaid on LB agar (containing 1.5% agar) [10]. Then the plates were incubated at 37 °C for 18 h and were examined for the presence of lysis zone or for plaques. The plaques were transferred to a nylon membrane (Hybond- N+, Amersham Pharmacia Biotech) according to the standard procedure [30] and hybridized at 65 °C with digoxigenin (DIG)-labelled *stx*_2_ probe. To prepare the *stx*_2_ probe, a 372-bp DNA fragment of the *stx*_2_ gene was purified by using a DNA purification kit (Qiagen Kit) and the purified product was labeled with DIG according to the manufacturer’s instructions (Roche Diagnostics, Barcelona, Spain). Stringent hybridization was carried out with the DIG–DNA Labelling and Detection kit (Roche Diagnostics) according to the manufacturer’s instructions.

### Construction of lysogens

To assess the lysogenization ability of *stx*_2_ phages we used different bacterial strains, including *E. coli* MC1061*, S. sonnei* and *E. coli* DH5α. Three hundred microlitres of an exponential-phase culture of host strain was added to 30 μl of 1 M CaCl_2_ and 6 ml molten LB top agar (LB broth with 0.7% agar); the mixture was then poured onto LB agar plates and allowed to solidify. Twenty microlitres of a suspension of each phage lysate was spotted onto the plates. After overnight incubation at 37 °C, the LB soft agar overlay was removed and resuspended in 1 ml SM buffer [10 mM Tris-Cl (pH 7.5), 10 mM MgCl_2_, 100 mM NaCl, 0.1% Gelatin] [30]. The mixture was incubated for 90 min at 37 °C, and then 0.1 ml portions of 10-fold dilutions in phosphate-buffered saline were plated onto LB agar. The plates were incubated at 37 °C for overnight. Lysogeny of bacterial hosts carrying the *stx*_2_ gene were identified by colony hybridization method as described previously [31] with *stx*_2_ probe used in the plaque blot hybridization. The presence of *stx*_2_ genes in lysogenic bacteria was confirmed by PCR. The production of Stx2 by the lysogenic strains was tested using a commercially available RPLA kit (VTEC-RPLA, Oxoid ltd, UK). Finally, PFGE of *Xba*I digested total chromosomal DNA of lysogenic strains were done along with host strain (*E. coli* MC1061) and Stx2-producing isolate (G51) from which bacteriophage was isolated. PFGE banding patterns of the lysogenic strain were compared with that of the corresponding non-lysogenic host strain to determine possible changes in the band-patterns due to integration of phage DNA into the chromosome of the host strain. To further confirm the integration of phage DNA in the chromosome of the host strain, relevant bands were excised from PFGE gel and the DNA was extracted by Montage Gel Extraction Kit (Merck Millipore) according to manufacturer’s instructions. The extracted DNA was then subjected to PCR assays for *stx*_2_ gene to test for the presence of the phage genome.

## Results

### Majority of *E. coli* O157 isolates are non-toxigenic despite carrying virulence genes

Despite being positive for *stx*_2_ gene, 42 out of 47 *E. coli* O157 (89%) produced no (Stx2 titre: < 2) or lower level of toxin (Stx2 titre: 8 to 32) and the remaining 5 isolates (11%) produced high titre of Stx2 toxin (Stx2 titre: 64 to 128) in VTEC RPLA assay [32]. Of the 5 isolates, 2 and 3 isolates were obtained from goat feces and buffalo feces, respectively (Table 1). None of the isolates except for one (C35) were positive for *stx*_1_ gene, and all isolates were positive for other virulence genes including *eae, katP, etpD and hly*_EHEC_ genes. All isolates were negative for *cdt* gene. Subtyping of *stx*_2_ gene revealed that 46 of 47 isolates were carrying both *stx*_2a_ and *stx*_2d_ and remaining isolate was positive for *stx*_2d_ only. All isolates were negative for *stx*_2b_, *stx*_2c_, *stx*_2e_, *stx*_2f_ and *stx*_2g_.

**Table 1:** Characteristics of Shiga toxin-producing *E. coli* O157 isolated from food and food-producing animals

ID no.	Source	*stx*_2_^a^	Stx_2_ titre	*cdt* ^a^	TNP^a^				NM^a^ PCR	*Q*_933_/*Q*_21_^d^	Presence of *stx*_2_ subtypes	Phage integration site^e^	
					A	B	C	D				*yehV*	*wrbA*
C8	Cow	+	-^b^	–	+	+	+	+	+	*Q*_21_	*stx*_*2a*_ *, stx*_*2d*_	I	I
C21	Cow	+	–	–	+	+	+	+	+	*Q*_21_	*stx*_*2a*_ *, stx*_*2d*_	I	I
C35	Cow	+	–	–	+	+	+	+	+	*Q*_21_	*stx*_*2a*_ *, stx*_*2d*_	I	I
C61	Cow	+	–	–	+	+	+	+	+	*Q*_21_	*stx*_*2a*_ *, stx*_*2d*_	I	I
C62	Cow	+	2	–	+	+	+	+	+	*Q*_21_	*stx*_*2a*_ *, stx*_*2d*_	I	I
C96	Cow	+	–	–	+	+	+	+	+	*Q*_21_	*stx*_*2a*_ *, stx*_*2d*_	I	I
C106	Cow	+	–	–	+	+	+	+	+	*Q*_21_	*stx*_*2a*_ *, stx*_*2d*_	I	I
C111	Cow	+	–	–	+	+	+	+	+	*Q*_21_	*stx*_*2a*_ *, stx*_*2d*_	I	I
C118	Cow	+	–	–	+	+	+	+	+	*Q*_21_	*stx*_*2a*_ *, stx*_*2d*_	I	I
M2	Buffalo	+	–	–	+	+	+	+	+	*Q*_21_	*stx*_*2a*_ *, stx*_*2d*_	Variant-R	I
M18	Buffalo	+	64	–	+	+	+	+	+	*Q*_21_	*stx*_*2a*_ *, stx*_*2d*_	I	I
M21	Buffalo	+	–	–	+	+	+	+	+	*Q*_21_	*stx*_*2a*_ *, stx*_*2d*_	I	I
M29	Buffalo	+	–	–	+	+	+	+	+	*Q*_21_	*stx*_*2a*_ *, stx*_*2d*_	I	I
M34	Buffalo	+	–	–	+	+	+	+	+	*Q*_21_	*stx*_*2a*_ *, stx*_*2d*_	I	I
M42	Buffalo	+	–	–	–	+	+	+	+	*Q*_21_	*stx*_*2a*_ *, stx*_*2d*_	I	I
M52	Buffalo	+	–	–	–	–	–	–	+	*Q*_21_	*stx*_*2a*_ *, stx*_*2d*_	I	I
M58	Buffalo	+	–	–	+	+	+	+	+	*Q*_21_	*stx*_*2a*_ *, stx*_*2d*_	I	I
M64	Buffalo	+	2	–	+	+	+	+	+	*Q*_21_	*stx*_*2a*_ *, stx*_*2d*_	I	I
M98	Buffalo	+	–	–	+	+	+	+	+	*Q*_21_	*stx*_*2a*_ *, stx*_*2d*_	I	I
M103	Buffalo	+	2	–	+	+	+	+	+	*Q*_21_	*stx*_*2a*_ *, stx*_*2d*_	I	I
M112	Buffalo	+	8	–	+	+	+	+	+	*Q*_21_	*stx*_*2a*_ *, stx*_*2d*_	I	I
M126	Buffalo	+	2	–	+	+	+	+	+	*Q*_21_	*stx*_*2a*_ *, stx*_*2d*_	I	I
M129	Buffalo	+	32	–	+	+	+	+	+	*Q*_21_	*stx*_*2a*_ *, stx*_*2d*_	Variant-R	I
M133	Buffalo	+	128	–	+	+	+	+	+	*Q*_21_	*stx*_*2a*_ *, stx*_*2d*_	I	I
M139	Buffalo	+	–	–	+	+	+	+	+	*Q*_21_	*stx*_*2a*_ *, stx*_*2d*_	I	I
M143	Buffalo	+	32	–	+	+	+	+	+	*Q*_21_	*stx*_*2a*_ *, stx*_*2d*_	I	I
M163	Buffalo	+	128	–	+	+	+	+	+	*Q*_21_	*stx*_*2a*_ *, stx*_*2d*_	I	I
M168	Buffalo	+	2	–	+	+	+	+	+	*Q*_21_	*stx*_*2a*_ *, stx*_*2d*_	I	I
M171	Buffalo	+	16	–	+	+	+	+	+	*Q*_21_	*stx*_*2a*_ *, stx*_*2d*_	Variant-R	I
M173	Buffalo	+	16	–	+	+	+	+	+	*Q*_21_	*stx*_*2a*_ *, stx*_*2d*_	I	I
G10	Goat	+	2	–	+	+	+	+	+	*Q*_21_	*stx*_*2a*_ *, stx*_*2d*_	I	I
G51	Goat	+	64	–	+	+	+	+	+	*Q*_21_	*stx*_*2a*_ *, stx*_*2d*_	Variant-R	I
G56	Goat	+	–	–	–	+	+	–	+	–	*stx*_*2a*_ *, stx*_*2d*_	O	I
G61	Goat	+	2	–	+	+	+	–	+	–	*stx*_*2a*_ *, stx*_*2d*_	Variant-R	I
G63	Goat	+	16	–	+	+	+	–	+	–	*stx*_*2a*_ *, stx*_*2d*_	Variant-R	I
G67	Goat	+	–	–	+	+	+	+	+	*Q*_21_	*stx*_*2a*_ *, stx*_*2d*_	O	I
G71	Goat	+	128	–	+	+	+	–	+	–	*stx*_*2a*_ *, stx*_*2d*_	Variant-R	I
G72	Goat	+	2	–	+	+	+	+	+	*Q*_21_	*stx*_*2a*_ *, stx*_*2d*_	I	I
G85	Goat	+	2	–	+	+	+	+	+	*Q*_21_	*stx*_*2a*_ *, stx*_*2d*_	I	I
G99	Goat	+	–	–	+	+	+	+	+	*Q*_21_	*stx*_*2a*_ *, stx*_*2d*_	I	I
CM2	Beef	+	–	–	+	+	+	+	+	*Q*_21_	*stx*_*2a*_ *, stx*_*2d*_	I	I
CM5	Beef	+	–	–	+	+	+	+	+	*Q*_21_	*stx*_*2a*_ *, stx*_*2d*_	O	I
CM7	Beef	+	–	–	+	+	+	+	+	*Q*_21_	*stx*_*2a*_ *, stx*_*2d*_	O	I
CM52	Beef	+	–	–	+	+	+	+	+	*Q*_21_	*stx*_*2a*_ *, stx*_*2d*_	I	I
CM56	Beef	+	–	–	+	–	+	+	–	–	*stx*_*2a*_ *, stx*_*2d*_	I	I
MM27	Buf Meat^c^	+	–	–	+	+	+	+	+	*Q*_21_	*stx*_*2a*_ *, stx*_*2d*_	I	I
MM28	Buf Meat^c^	+	–	–	+	–	+	+	–	–	*stx*_*2d*_	I	I
*E. coli* NCTC12079	Clinical isolate	+	128	–	–	–	–	–	–	*Q*_933_	ND^f^	ND^f^	

^a^Examined by PCR method. +, positive; −, negative

^b^Stx2 toxin detection. -, not detected

^c^Buffalo meat

^d^Presence of either *Q*_933_ or *Q*_21_ gene. -, absence of both genes

^e^I, intact; O, occupied. ‘Intact’ indicates detection of neither junction. ‘Variant-R’ indicates that the left junction between the bacteriophage and the chromosome was detected but the right junction was not detected. ‘Occupied’ indicates bilateral detection of that integration site

^f^Not Done

### PCR assays demonstrated that *E. coli* O157 isolates carry non-functional promoter sequence in the *stx* region

Thirty nine out of 47 isolates were positive for all four genes specific for TNP region (Table 1). Of the remaining 8 isolates, 7 were positive for either one or three out of 4 genes and one isolate was negative for all 4 genes. Presence of all 4 TNP genes indicates that the isolates have a non-functional promoter sequence in the Stx region [23]. Forty five out of 47 isolates were positive for NM specific PCR. Forty-one of 47 isolates were positive for *Q*_21_ gene. All the 5 isolates that were negative for *Q*_21_ gene were partially negative for TNP genes. None of the 47 isolates were positive for *Q*_933_ gene (Table 1).

### *yehV* and *wrbA* phage insertion sites were intact in majority of *E. coli* O157 isolates

Of the 47 *E. coli* O157 isolates, 11 did not produce any amplicon for *yehV* gene, indicating that this site maybe occupied by a bacteriophage (Table 1). Insertion of phages in this site was further confirmed by detecting regions spanning from left and right junctions of the insertion to bacteriophages. Of the 11 isolates, four were positive for amplification of the left and right *yehV-*bacteriophage junction and the remaining seven isolates were positive for the left but not the right *yehV-*bacteriophage junction. All isolates had amplification of *wrbA* gene indicating that this site is intact in all the isolates. Intact *wrbA* site was further confirmed by the absence of any amplification in the left and right *wrbA*-bacteriophage junction.

### Only toxin-producing isolates carried inducible *stx* phages

**Fig. 1:**
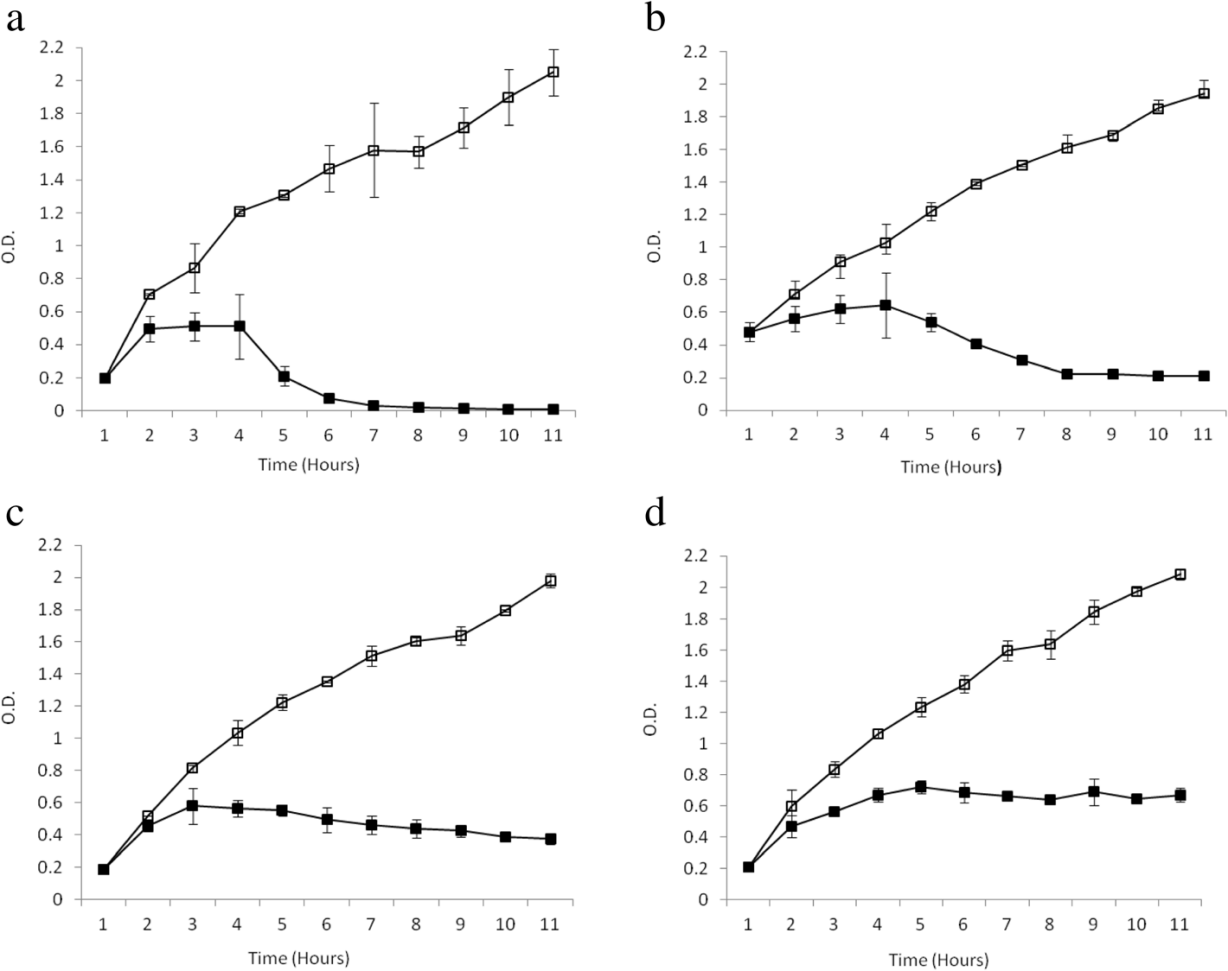
Kinetics of phage induction in toxin-producing and toxin non-producing *E. coli* O157 isolates. Two toxin-producing *E. coli* O157 strains (**a** M163; **c** M18) along with one toxin negative *E. coli* O157 strain (**d** M103) and positive control strain *E. coli* O157:H7 (**b** NCTC 12079) were used. Each culture was grown to an opitical density (OD) of 0.2 at 600 nm and then separated into two aliquots, one was treated with mitomycin C (■) and the other one was not (□) and bacterial cell growth in both aliquots were followed at every 1 h interval upto 11 h. OD data (Y-axis) were plotted against time in h (X-axis). Error bars depict standard errors of the means of three replicas

Growth kinetics of isolates varied from each other when cells were induced with MMC. Some isolates contained phages that were inducible and the level of induction varied from isolate to isolate (Fig. 1). Significant reduction of growth of M163 isolate was observed when cultured with MMC as compared to growth without MMC (Fig. 1a). This is comparable with the growth pattern of the positive control strain *E. coli* O157:H7 NCTC 12079 (contain both *stx*_1_ and *stx*_2_ gene) (Fig. 1b). For both isolates, when culture was done with MMC, multiplication of cells occurred at same level up to the first 2 h and cell growth was stationary at that level for the next 2 h followed by rapid decline of growth within next 1 h. In the case of MMC free culture, cell growth kept on rising at the same rate. At 7 h point, OD (at 600 nm) of MMC added culture came down to zero while in case of culture without MMC, it increased up to 1.5. In the case of M18, M133, G51 and G71, bacterial growth in presence of MMC was also slowed down as compared to growth without MMC but the complete cessation of growth was not observed (Fig. 1c). In contrast, for toxin non-producing isolate M103, cell growth increased in the first 5 h for MMC added culture then became relatively stable, although cell growth increased at a normal rate for MMC free culture (Fig. 1d).

### *stx*_2_ phages carried by Shiga toxin-producing *E. coli* O157 isolates are genetically clustered

**Fig. 2:**
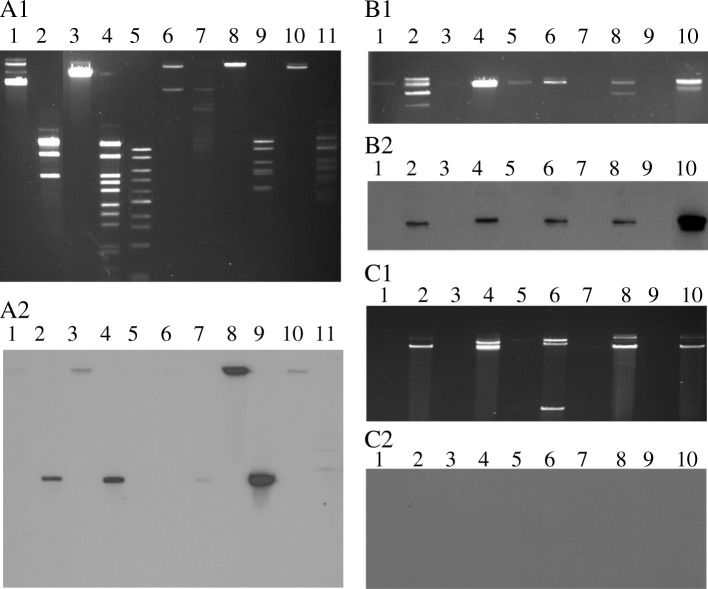
Field inversion gel electrophoresis (FIGE) analysis of phage DNA. (A1) FIGE analysis of *Eco*RI- digested phage DNA induced from toxin-producing *E. coli* O157 isolates and (A2) its corresponding blot hybridized with radioactively labeled *stx*_2_ probe. Lane 1: M18 (undigested), Lane 2: M18 (digested), Lane 3: M133 (undigested), Lane 4: M133 (digested), Lane 5: 1 kb Marker, Lane 6: M163 (undigested), Lane 7: M163 (digested), Lane 8: G51 (undigested), Lane 9: G51 (digested), Lane 10: G71 (undigested), Lane 11: G71 (digested). (B1) FIGE analysis of phage DNA induced and uninduced from toxin-producing *E. coli* O157 isolates and (B2) its corresponding blot hybridized with radioactively labeled *stx*_2_ probe. Lane 1: M18 (uninduced), Lane 2: M18 (induced), Lane 3: M133 (uninduced), Lane 4: M133 (induced), Lane 5: G71 (uninduced), Lane 6: G71 (induced), Lane 7: M163 (uninduced), Lane 8: M163 (induced), Lane 9: G51 (uninduced), Lane 10: G51 (induced). (C1) FIGE analysis of phage DNA induced and uninduced from *E. coli* O157 isolates producing little or no toxin and (C2) its corresponding blot hybridized with radioactively labeled *stx*_2_ probe. Lane 1: C96 (uninduced), Lane 2: C96 (induced), Lane 3: M143 (uninduced), Lane 4: M143 (induced), Lane 5: M168 (uninduced), Lane 6: M168 (induced), Lane 7: M171 (uninduced), Lane 8: M171 (induced), Lane 9: M173 (uninduced), Lane 10: M173 (induced)

Field Inversion Gel Eelectrophoresis (FIGE) analysis of phage DNA revealed diversity among phages induced from different *E. coli* O157 isolates. Based on the number of bands detected in FIGE analysis of undigested phage DNA, we found that all the four TNP positive isolates that produce high titre of toxins (M18, M133, M163 and G51) carried more than one bacteriophage. Digestion of phage DNA with *Eco*R1 restriction enzyme produced multiple bands (4 to 14) and banding patterns were different from one phage to another (Fig. 2A1). Digested phage DNA was transferred to nylon membrane and hybridized with radioactively labeled *stx*_*2*_ probe in order to specify the location of *stx*_2_ gene within phage DNA. Positive signals were found in case of four phages, where *stx*_2_ probe was hybridized with a single band of 5.5 kb in size. G71, the TNP negative strain that produce high titre of toxin, also carried more than one phages and *stx*_2_ probe was hybridized with a single band of 6 kb in size (Fig. 2A2). The hybridization results indicate that all of the five *E. coli* O157:H7 strains carry non-stx phages in addition to *stx* phages.

Phage DNA extracted from *E. coli* isolates having different toxin-producing capacity [high (≥64), low (≤32) or no titre (< 2) of toxins] with and without MMC induction were analysed by FIGE to determine if there is any association between toxin production and inducibility of phages. Considerable increase in intensity of *stx*_2_ phage DNA (and thus replication) was observed after MMC induction of isolates that produced relatively high titre of toxin (Fig. 2B1). Presence of *stx*_2_ in these phages was confirmed by PCR and hybridization with *stx*_2_ gene probe (Fig. 2B2). Similar level of increase in phage DNA intensity was also observed in case of little or no toxin producing isolates (C96, M143, M168, M171, M173) (Fig. 2C1) but these inducible phages were not *stx*_2_-carrying as none of phage DNA gave positive signal in Southern hybridization with *stx*_2_ probe (Fig. 2C2).

### *stx*_2_ phages induced from STEC O157 isolates can infect diverse host strains

The ability of induced *stx*_2_-phages to infect different hosts was analysed by plaque-forming assay and by hybridization of the plaques with a 372-bp *stx*_2_ gene probe (Table 2). All *stx*_2_-phages induced from STEC originated from buffalo (M18, M133, M163) infected clinical isolate of *S. sonnei* but the phages induced from isolates originated from goat (G51, G71) could not infect *S. sonnei*. *E. coli* MC1061, DH5α and *S. dysenteriae* 4 were infected by three phages each (Table 2). Phage induced from M133 infected *E. coli* O157:H7 NCTC 12900 (Stx negative), while phage induced from the control *E. coli* O157:H7 NCTC 12079 isolate infected *E. coli* MC1061 and *S. sonnei*. Although clear infection of *S. dysenteriae* type 2 (data not shown) and type 4 was observed with phages from M163 and G71 isolates, respectively but these plaques were negative in plaque blot hybridization assay with *stx*_2_ probe suggesting that isolates carrying inducible phages other than *stx*_2_ phage.

**Table 2:** Infectivity of toxin-producing *stx*_2_ phages in different bacterial host

Bacterial species	Phage ID number					
	Ф12079	ФM133	ФM163	ФG71	ФM18	ФG51
*Shigella sonnei*	+	+	+	–	+	–
*E. coli* MC1061	+	–	+	+	–	+
*E. coli* DH5α	–	–	+	+	–	+
*S. dysenteriae* type 4	–	+	–	-^a^	+	–
*E. coli* O157:H7 NCTC12900	–	+	–	–	–	–

+, Strong signal; −, no signal after hybridization of the plaque with the specific *stx*_2_-probe

^a^ФG71 produced clear plaques on culture lawn of *S. dysenteriae* type 4 but these plaques did not give positive signal in plaque blot hybridization assay with *stx*_2_-probe

### *stx*_2_ phage induced from one STEC O157 isolate lysogenized different bacterial strains

**Fig. 3:**
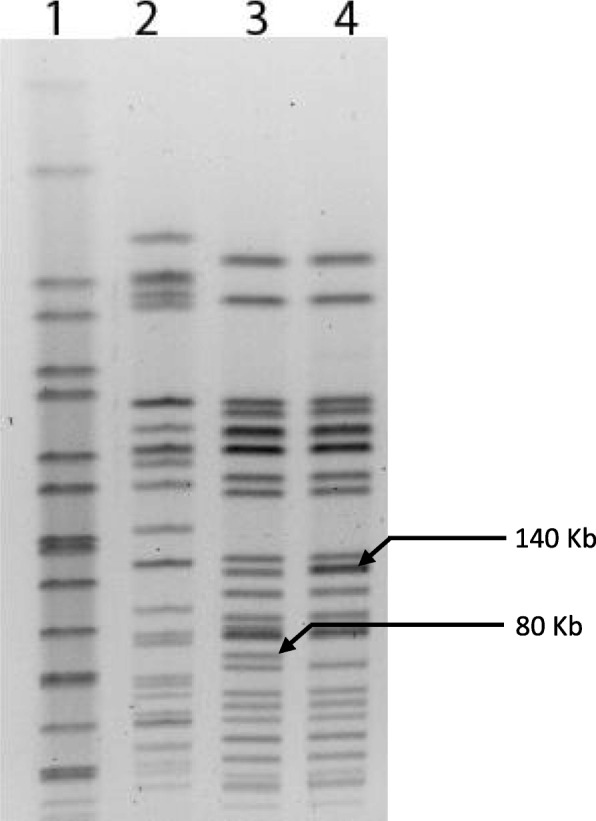
Detection of integration of *stx*_2_ phage DNA with *E. coli* MC1061 chromosome by PFGE analysis. Lane 1: *Salmonella* Braenderup, Lane 2: *E. coli* O157 G51, Lane 3: *E. coli* MC1061, Lane 4: *E. coli* MC1061-Lysogen. The bottom arrow mark denotes the position of the band in *E. coli* MC1061 which is missing in all lysogenic strains of *E. coli* MC1061. The arrow mark above denotes the intensity of a band in all lysogenic strains of *E. coli* MC1061 which is significantly higher than that of the non-lysogenic *E. coli* MC1061 strain

The ability of *stx*_2_ phages to infect and lysogenize different susceptible bacterial strains was assessed. Phage induced from isolates G51 successfully lysogenized *E. coli* MC1061, which was stable after repeated sub-cultivation of the transduced strain. The lysogenized strain produced high titre of Stx2 toxin in VTEC-RPLA assay (toxin titre 64). None of the phages from remaining isolates was found to lysogenize bacterial hosts. PFGE banding pattern of phage integrated host strain was almost identical with that of phage non-integrated strain except for disappearance of an 80 kb band in the lysogenized strain along with the intensification of a 140 kb band, which is indicative of the integration of phage DNA into bacterial host chromosome (Fig. 3). For further confirmation of integration, we excised the intensified band of lysogenic strain and its corresponding band in host strain (*E. coli* MC1061), extracted DNA from the gel and did PCR for *stx*_2_ gene. We also excised the 80 kb band from the host strain and did PCR for *stx*_2_ gene using template DNA extracted from gel. Only the intensified 140 kb band from lysogen was found positive for *stx*_2_ gene while none of the 140 kb and 80 kb bands in host strain (*E. coli* MC1061) was positive demonstrating the integration of *stx*_2_ gene in the bacterial chromosome.

## Discussion

Previous studies have demonstrated that *E. coli* O157 isolates of cattle origin, particularly from Asian countries, contain defective *stx* phages and, therefore, cannot produce sufficient amounts of toxin required to cause severe human infection [23]. In this study, we found that the majority (89%) of *E. coli* O157 isolates from food or food-producing animal (cattle) origin have little or no ability to produce Shiga toxin in spite of being positive for the *stx* gene. This could be one of the reasons for a low prevalence of *E. coli* O157 infection in humans in Bangladesh [33]. As the most important virulence factor of *E. coli* O157 is Stx2 and adhesin intimin; the role of other virulence factor such as enterohaemolysin, a serine protease (EspP) and catalase or peroxidase (KatP) in causing infection in human may be low [34]. Lower prevalence of *E. coli* O157 infection in humans have also been reported from other Asian countries including Thailand and Malaysia [18, 19]. However, unlike Thailand and Malaysian studies, we found that a number of isolates (*n* = 5; 11%) can produce high titre of Stx2 using the VTEC-RPLA assay. We analysed these isolates for a set of four PCR-amplifiable genetic loci as markers that were previously reported to be linked with toxin non-producing (TNP) characteristics of *E. coli* O157. We found that most of the isolates were positive for these four genetic markers whereas a few isolates showed negative results for one or two of these markers. Among the 5 isolates that were capable of producing high titre of toxins, four were positive for TNP PCR, which is not in agreement with the previous study [23]. Although in that study [23], a small number of TNP positive isolates were found to produce Stx, the level of toxin production was much lower than that of Stx positive and TNP negative isolates. Subsequently, in another study, a high number of TNP positive human isolates were found to produce a high titre of Stx [24]. Therefore, the application of TNP PCR for detecting toxin non-producing isolates needs further evaluation. Considering this pitfall of TNP PCR, Taylor et al. [24] recommended that detection of bacteriophage antiterminator gene alleles *Q*_933_and *Q*_21_ could be a better marker for the detection of TNP strains from both human and animal origins. Previous studies demonstrated that *E. coli* O157:H7 strains harboring the antiterminator gene variant *Q*_933_ produced significantly higher levels of Stx2 than strains with the *Q*_21_ variant [12]. None of the isolates in the present study was positive for *Q*_933_, although 5 isolates produced moderate to high level of Stx, in contrast with the previous study [12]. All of these four isolates producing high titre Shiga toxin were positive for *Q*_21_, which is also not in agreement with previous reports [12, 35], although a majority of toxin negative isolates were found positive for *Q*_21_. In sum, none of the PCR methods developed to date is 100% specific for detecting toxin non-producing isolates and thus toxin assay is still the most reliable approach.

Typing of Stx2 provides important epidemiological characteristics of STEC isolates. In this study, we found that all isolates, except for one, carried a combination of *stx*_2a_ and *stx*_2d_ subtypes. STEC strains carrying more than one *stx*_2_ subtypes have been reported in previous studies [25, 36]. According to previous study, the combination of Stx2a and Stx2d were more potent than Stx2b, Stx2c and Stx1 in both cell culture and animal models [37]. However, epidemiological data suggest that STEC O157 strains carrying Stx2a and Stx2c are most commonly associated with HUS in humans [36, 38, 39].

Analysis of phage integration sites in *E. coli* O157 showed that only a few isolates have Stx2 phage integration sites at *yehV* and all isolates have intact *wrbA* site, which is inconsistent with previous study where *wrbA* was found to be the most commonly occupied site by Stx2 encoding bacteriophages [27]. Nevertheless, some studies suggested that *yehV* is commonly occupied by bacteriophages in *E. coli* O157 isolated from cattle [40]. It has also been reported that strains carrying *stx*_2_ phages at *yehV* site were amplification-positive for the left but not the right *yehV* junction, a characteristic more commonly seen in cattle isolates compared to the clinical isolates [41]; corroborating with isolates in this study. Nonetheless, phage occupancy was not detected in either of *yehV* and *wrbA* sites in a significant number of isolates, accentuating the need for further exploration of isolates for the presence of other insertion sites.

In order to get more insight into characteristics of TNP *E. coli* O157 isolates, we examined the inducibility of *stx* phages present in these isolates. Apart from being the reservoir of main virulence factors of STEC, *stx* phages are involved in the regulation of pathogenicity factors and the development of genomic plasticity of host bacteria [42]. Among virulent *E. coli* O157 strains, high titre of toxin production is coupled with the production of infectious phage particles [23]. TNP isolates cannot produce a high titre of toxin due to the poor expression of late phage genes from the *p*_*R’*_ promoter [23]. However, in our study we found that four TNP PCR positive isolates were able to produce complete phage particles after MMC induction. For the induction assay, we used *E. coli* O157:H7 12,079 as a control which carries MMC inducible *stx* phage. Complete cessation of bacterial growth was observed in this isolate within 10 h of MMC treatment. Comparing the degree of induction of phages in *E. coli* O157:H7 12,079 with phages in cattle isolates, we found that phages in M163 had the same degree of induction with *E. coli* O157:H7 12,079 strain while phages in M133, M18, G51 and G71 were inducible at a relatively slower rate (Fig. 1).

PCR confirmed the presence of *stx*_2_ gene in induced phage DNA extracted from all five isolates. *Eco*R1 digestion of phage DNA revealed a high degree of heterogeneity among DNA bands in FIGE indicating a considerable diversity of phage genomes. However, the heterogeneity was not seen in hybridization of *stx*_2_ gene as a single band of similar size was found to be hybridized with most of the phage DNA samples. The heterogeneity in *Eco*R1 digested phage DNA might be due to the presence of non-*stx* phages present in the isolates.

Horizontal gene transfer plays a major role in virus evolution by creating new combinations of genetic material through pairing and shuffling of related DNA sequences [42–44]. We have shown that the phages induced from 5 STEC isolates could infect a range of bacterial isolates such as *E. coli* DH5α, *E. coli* MC1061, clinical isolates of *Shigella sonnei* and *S. dysenteriae* type 4. More precisely, 3 of the 5 *stx*_2_ phages could infect a clinical isolate of *S. sonnei*. *Stx* gene containing bacteriophage in *S. sonnei* was also reported previously and studies suggested that *Shigella* might play a role in spreading of *stx* genes [45–48]. This finding has important public health implication in Bangladesh given that the prevalence of *S. sonnei* has been increasing in patients with diarrhoea in the country [49]. A new variant of *S. sonnei* has been recently emerged, which carries all essential virulence genes needed for enteroinvasive pathogenesis along with an inducible Stx1 encoding prophage providing additional virulence potential related to Shiga toxin [50].

Among six bacteriophages used in testing lysogenic conversion of recipient bacterial strains, we identified lysogens of *stx*_2_ phage induced from isolate G51 in *E. coli* MC1061. The lysogenized strain produced a high titre of toxin similar to parent strain without MMC induction. In PFGE analysis, *Xba*I digestion of chromosomal DNA of lysogenic and non-lysogenic strains of *E. coli* MC1061 confirmed the integration of phage DNA to MC1061 DNA. We found that a single band of approximately 80 kb disappeared from lysogenic strains while another band of approximately 140 kb in size substantially intensified, suggesting that an additional band of similar size is present in the same position (Fig. 3). The *stx*_2_ phage DNA has genome sizes in the range from 49 to 66 kb [10, 51]. The integration of phage genome with bacterial chromosome at the site of 80 kb band results in increase of the band size in the range of 129 kb to 146 kb and this is what has been identified in PFGE- the increase of intensity of 140 kb band in *E. coli* MC1061 (Fig. 3). In general, *stx* lysogens occur at a low frequency and all *stx* phages are not capable of generating lysogens at the same frequency [52] or lysogenize the same bacterial host strain [53]. *stx* phages induced from lysogenic strains generate lysogen rarely even under the best conditions [41].

Nearly all bacterial genomes comprise of multiple active or defective phages [54]. Prophages and integrative elements constitute about 30% genome of STEC strains [55]. For temperate phages, prophage remnants, entrapped in host chromosome, can be a receptacle of functional lytic cycle genes [54]. Similarly, defective prophages which were known as simple genetic junk or remnants, are now recognized for a wide range of characteristics that are essential to bacterial hosts with respect to virulence, stress resistance, or even the rate of mutation [28, 55–57]. Environmental factors are known to influence the induction and transmissibility of lysogenic phages and the biology of bacterial strains carrying such phages. In view of predicted climatic changes likely to adversely affect various regions of the world, in particular, the low-lying Ganges delta region, marked changes in the ecology, human behavior and epidemiology of infectious diseases are likely to occur. Therefore, human pathogens which are abundant in food-producing animals may pose considerably enhanced risks of human infection under the changed settings. This accentuates further investigation of defective phages in *E. coli* O157 isolates producing no or low toxin, particularly in Asian countries where the prevalence of these organisms in the animal reservoir is high. Besides, detection of active and infectious phages with lysogenic properties in the current and previous [28, 55] studies indicated that phage mediated transfer of Stx genes from pathogenic to non-pathogenic strains can readily occur in the environment, particularly in areas polluted with animal feces carrying STEC. Our results warrant further investigations to monitor the emergence and evolution of Shiga toxin-producing organism with relevance to public health.

## Conclusions

The majority of *E. coli* O157 from food and food-producing animals tested in this study are not capable of producing Shiga toxin, the main virulence factor for the organism. This might be one of the reasons that *E. coli* O157 infection is not prevalent in humans in Bangladesh. The non-toxigenic characteristic of *E. coli* O157 strains is attributed to the presence of genetically defective *stx* phages in isolates. Unlike non-toxigenic isolates, the study also detected a few toxin-producing isolates carrying active *stx* phage. *Stx* phages from these isolates are capable of infecting multiple bacterial hosts and producing stable lysogen in *E. coli* and thus converting it into a toxigenic strain. This implies that the evolution of new STEC can readily occur in the environment, particularly in areas polluted with cattle feces.

## Abbreviations

DIG: Digoxigenin
FIGE: Field inversion gel electrophoresis
LB: Luria Bertani
MMC: Mitomycin C
OD: Optical density
PEG: Polyethylene glycol
PFGE: Pulsed field gel electrophoresis
RFLP: Restriction fragment length polymorphism
RPLA: Reverse passive latex agglutination assay
STEC: Shiga toxin-producing *E. coli*
Stx: Shiga toxin
Stx1: Shiga toxin 1
Stx2: Shiga toxin 2
TNP: Toxin non-producing
